# Supplementary material for: Dynamics of cortical contrast adaptation predict perception of signals in noise
Source: Nat Commun. 2023 Aug 9;14:4817. doi: 10.1038/s41467-023-40477-6 (PMC10412650; doi:10.1038/s41467-023-40477-6)
Supplement: Supplementary file 2 — Reporting Summary [file 41467_2023_40477_MOESM2_ESM.pdf]

nature research

Corresponding author(s): Maria N. Geffen

Last updated by author(s): May 25, 2023

Reporting Summary

Nature Research wishes to improve the reproducibility of the work that we publish. This form provides structure for consistency and transparency in reporting. For further information on Nature Research policies, see our Editorial Policies and the Editorial Policy Checklist.

Statistics

For all statistical analyses, confirm that the following items are present in the figure legend, table legend, main text, or Methods section.

n/a	Confirmed	
☐	☒	The exact sample size (*n*) for each experimental group/condition, given as a discrete number and unit of measurement
☐	☒	A statement on whether measurements were taken from distinct samples or whether the same sample was measured repeatedly
☐	☒	The statistical test(s) used AND whether they are one- or two-sided<br>*Only common tests should be described solely by name; describe more complex techniques in the Methods section.*
☐	☒	A description of all covariates tested
☐	☒	A description of any assumptions or corrections, such as tests of normality and adjustment for multiple comparisons
☐	☒	A full description of the statistical parameters including central tendency (e.g. means) or other basic estimates (e.g. regression coefficient) AND variation (e.g. standard deviation) or associated estimates of uncertainty (e.g. confidence intervals)
☐	☒	For null hypothesis testing, the test statistic (e.g. F, t, r) with confidence intervals, effect sizes, degrees of freedom and P value noted<br>*Give P values as exact values whenever suitable.*
☒	☐	For Bayesian analysis, information on the choice of priors and Markov chain Monte Carlo settings
☒	☐	For hierarchical and complex designs, identification of the appropriate level for tests and full reporting of outcomes
☐	☒	Estimates of effect sizes (e.g. Cohen's *d*, Pearson's *r*), indicating how they were calculated

Our web collection on statistics for biologists contains articles on many of the points above.

Software and code

Policy information about availability of computer code

Data collection	All data collection hardware and software are described in Online Methods or Supplemental Methods in the manuscript. Briefly: electrophysiological data was acquired using either a Cheetah Digital Lynx system running Cheetah 5.7.4, or an openEphys Aquisition Board running openEphys Plugin GUI v0.4.3.3 with plugin API v5 or v0.4.5 with plugin API v6. Behavioral data was collected using a customized hardware and software system. An Arduino UNO microprocessor handled lick detection, reward delivery, collected event times and controlled low-level task logic and timing. Custom software written in MATLAB (r2015a-r2019a) controlled high-level task logic and stimulus generation, and logged task events sent from the Arduino via a USB port.
Data analysis	All data was analyzed using MATLAB (r2019a). For large scale simulations and model fitting, compiled MATLAB code (r2018a) was run in parallel on the CUBIC cluster at the University of Pennsylvania, controlled using BASH scripts (v3.2.57(1)). Electrophysiological data was sorted using Kilosort or Kilosort2, then manually curated using Phy or Phy2.<br><br>GitHub repositories for this paper can be found at:<br>https://github.com/geffenlab/contrast_glm<br>https://github.com/geffenlab/contrast_behavior

For manuscripts utilizing custom algorithms or software that are central to the research but not yet described in published literature, software must be made available to editors and reviewers. We strongly encourage code deposition in a community repository (e.g. GitHub). See the Nature Research guidelines for submitting code & software for further information.

Data

Policy information about availability of data

All manuscripts must include a data availability statement. This statement should provide the following information, where applicable:

- Accession codes, unique identifiers, or web links for publicly available datasets
- A list of figures that have associated raw data
- A description of any restrictions on data availability

All data including sorted spike times from electrophysiological recordings and behavioral data are available on DRYAD: https://doi.org/10.5061/dryad.6djh9w120.

Every Figure and Extended Data Figure will have associated data with the following exceptions. Figure 1, Extended Data Figure 1, and Extended Data Figure 2 are the results of simulations and thus have no associated raw data. Simulation parameters can be found in the code.

There are no restrictions on data availability.

Field-specific reporting

Please select the one below that is the best fit for your research. If you are not sure, read the appropriate sections before making your selection.

☒ Life sciences ☐ Behavioural & social sciences ☐ Ecological, evolutionary & environmental sciences

For a reference copy of the document with all sections, see nature.com/documents/nr-reporting-summary-flat.pdf

Life sciences study design

All studies must disclose on these points even when the disclosure is negative.

Sample size	No statistical methods were used to determine sample sizes, but we used 4-13 mice per experiment based on previous behavioral work studying signal in noise detection in mice (Tziridis et al, 2016; Chambers et al, 2016; Reznik & Polley, 2021) .
Data exclusions	Unless otherwise noted, behavioral sessions in which the false alarm rate exceeded 20% were discarded from analysis. One mouse (ID: CA122) had consistently high false alarm rates in the high contrast condition, so we excluded high contrast sessions from this mouse from all analyses. For Figures 5 and 6, we removed neurons with low spike rates (<1Hz) and noise-like or inverted (ie. upward inflected) spike waveforms. To determine waveform quality, we computed the width of each waveform at half of the minimum value (FWHM) and its correlation with the average waveform over all neurons. Neurons whose waveforms had outlier FWHM values (isoutlier in MATLAB), negative correlations, or were not significantly correlated with the average (Bonferoni corrected p > 5.85e-6) were removed from further analysis. For Figure 5f-l, sessions with stable population decoding performance were included (defined as sessions where more than half of the target types elicited significant population AUC values, as determined by the bootstrap procedure described previously). For Figure 6e-h, only neurons with noise ratios less than 100 were included in all analyses.
Replication	Experiments were typically performed using multiple cohorts of mice, with a typical cohort size of 4 trained every 2-3 months. We found that all of our results replicated across multiple, independent cohorts.
Randomization	In the behavioral task, mice were randomly counterbalanced to initialize target detection training in low contrast or high contrast. We found that mice in each group had a similar learning rate and reached comparable levels of stable performance (Figure 3b).
Blinding	Investigators were not blinded to the experimental conditions because: 1) investigators could hear the contrast of the stimulus during the initial training phase and, 2) to prevent cross-contamination of injection lines for muscimol and saline, investigator was aware of which solution was injected for a given session.

Reporting for specific materials, systems and methods

We require information from authors about some types of materials, experimental systems and methods used in many studies. Here, indicate whether each material, system or method listed is relevant to your study. If you are not sure if a list item applies to your research, read the appropriate section before selecting a response.

Materials & experimental systems

n/a	Involved in the study
☒	☐ Antibodies
☒	☐ Eukaryotic cell lines
☒	☐ Palaeontology and archaeology
☐	☒ Animals and other organisms
☒	☐ Human research participants
☒	☐ Clinical data
☒	☐ Dual use research of concern

Methods

n/a	Involved in the study
☒	☐ ChIP-seq
☒	☐ Flow cytometry
☒	☐ MRI-based neuroimaging

Animals and other organisms

Policy information about studies involving animals; ARRIVE guidelines recommended for reporting animal research

Laboratory animals

Adult (13-21 weeks) male (n = 19) and female (n = 19) mice (mus musculus) were used in this experiment. A subset of the mice were standard C57BL/6 strain (n=8), while the remainder were from CDH23 background strain to prevent age-related hearing degeneration (n=30). See Online Methods and Extended Data Table 2 for specific strains, genders and numbers used in each experiment. All mice were housed with, at most, five mice per cage, at 28°C on a 12-h light:dark cycle with food provided ad libitum, and a restricted water schedule. Ambient humidity was controlled to between 40-60%. All experiments were performed during the animals' dark cycle.

Wild animals

This study did not involve wild animals.

Field-collected samples

This study did not involve field-collected samples.

Ethics oversight

All experimental procedures were in accordance with NIH guidelines and approved by the Institutional Animal Care and Use Committee at the University of Pennsylvania.

Note that full information on the approval of the study protocol must also be provided in the manuscript.
